# Unmet health care needs: factors predicting satisfaction with health care services among community-dwelling Canadians living with neurological conditions

**DOI:** 10.1186/s12913-022-08611-0

**Published:** 2022-10-17

**Authors:** Tamara Chambers-Richards, Batholomew Chireh, Carl D’Arcy

**Affiliations:** 1 School of Health Sciences, College of New Caledonia, Prince George, British Columbia, Canada; 2 Saskatchewan Cancer Agency, 1804 McOrmond Drive, Saskatoon, SK Canada; 3 School of Public Health, University of Saskatchewan, Saskatoon, SK Canada; 4 Department of Psychiatry, University of Saskatchewan, Saskatoon, SK Canada

**Keywords:** Unmet needs, Predictors, Satisfaction, Neurological conditions, Canada

## Abstract

**Background:**

Neurological conditions (NCs) can lead to long-term challenges including functional impairments and limitations to activities of daily living. People with neurological conditions often report unmet health care needs and experience barriers to care. This study aimed to (1) explore the factors predicting patient satisfaction with general health care, hospital, and physician services among Canadians with NCs, (2) examine the association between unmet health care needs and satisfaction with health care services among neurological patients in Canada, and (3) contrast patient satisfaction between physician care and hospital care among Canadians with NCs.

**Methods:**

We conducted a secondary analysis on a subsample of the 2010 Canadian Community Health Survey - Annual Component data (N = 6335) of respondents with neurological conditions, who received general health care services, hospital services, and physician services within twelve months. Multivariate logistic regression fitted the models and odds ratios and 95% confidence intervals were reported using STATA version 14.

**Results:**

Excellent quality care predicts higher odds of patient satisfaction with general health care services (OR, 95%CI–237.6, 70.4–801.5), hospital services (OR, 95%CI–166.9, 67.9–410.6), and physician services (OR, 95%CI–176.5, 63.89–487.3). In contrast, self-perceived unmet health care needs negatively predict patient satisfaction across all health care services: general health care services (OR, 95%CI–0.59, 0.37–0.93), hospital services (OR, 95%CI–0.41, 0.21–0.77), and physician services (OR, 95%CI–0.29, 0.13–0.69). Other negative predictors of patient satisfaction include some post-secondary education (OR, 95%CI–0.36, 0.18–0.72) for general health services and (OR, 95%CI–0.26, 0.09–0.80) for physician services. Those with secondary (OR, 95% CI–0.32, 0.13–0.76) and post-secondary graduation (OR, 95%CI– 0.28, 0.11–0.67) negatively predicted patient satisfaction among users of physician services while being an emergency room patient most recently (OR, 95%CI– 0.39, 0.20–0.77) was also negatively associated with patients satisfaction among hospital services users.

**Conclusion:**

This study found self-perceived unmet health care needs as a significant negative predictor of neurological patients’ satisfaction across health care services and emphasizes the importance of ensuring coordinated efforts to provide appropriate and accessible care of the highest quality for Canadians with neurological conditions.

## Background

Neurological conditions (NCs) including Alzheimer’s disease (AD)/ dementia, Parkinson’s disease (PD), amyotrophic lateral sclerosis, sclerosis, and others, were the focus of a Statistics Canada survey in 2010 [1]. NCs, especially those exacerbated by increased age, e.g., PD and AD/dementia, lead to long-term challenges with functional impairments and limitations to activity [2]. Neurological patients, not surprisingly, report unmet health care needs [3, 4] and experience barriers to care including lack of resources (time and money), lack of services, and no local specialists [2, 5, 6].

Self-reported unmet health care need is a commonly used measure of health care access or utilization [7]. Health care utilization factors include availability, acceptability, accessibility, and personal choice (unrelated to the health system) [8, 9]. Perceived unmet health care needs may be categorized per availability – waiting time too long, care not available when requested, care not available in the area; acceptability – dislike doctor/afraid, language problems, didn’t know where to go; accessibility –cost and transportation; or personal choice – too busy, didn’t get around to it/didn’t bother, felt it would be inadequate, decided not to seek care, and personal/family responsibilities [6].

Anderson’s health behavior model describes health care utilization as a function of three factors: predisposing, enabling, and need. Predisposing factors exist before presentation with a health condition, i.e., socio-demographic or socio-cultural characteristics; enabling factors represent the logistical means for accessing health services; and need factors are the effectual cause of health service use and reflect the perceived health status of the health care user [10, 11]. The outcome measure for this study, patient satisfaction, is widely accepted as an assessment of overall healthcare quality [12, 13]. Patient satisfaction is associated with health-related quality of life (an individual’s or a group’s perceived physical and mental health over time) [14]. Some studies indicate that unmet health care needs result in decreased patient satisfaction with health care services [15–17] and lowered quality of health care and life [18–20].

Neurological conditions are a major contributor to disability in the Canadian population. Approximately 3.77 million Canadians live with neurological conditions. Of this number, 170,000 are cared for in institutions [21]. People with psychosocial difficulties, common to neurological conditions, have reported higher numbers of unmet health care needs [22–24] that may go unnoticed by health professionals [25]. Therefore, an understanding of unmet health care needs and patient satisfaction among older Canadians with NCs is crucial to the ongoing evaluation and continuous quality improvement of care for this vulnerable population [10]. Such knowledge will contribute to the health system’s preparation and strengthening of services to adequately meet the needs of the increasing aging population. This study examines the association between unmet health care needs and satisfaction with health care services in Canada among neurological patients. We incorporate life satisfaction as a predisposing factor of patients’ satisfaction with the health care system as it presents an overarching view of an individual’s satisfaction and may influence one’s satisfaction with the health system. The specific objectives of this study are (1) to explore the factors predicting patient satisfaction with general health care, hospital, and physician services among Canadians with NCs, (2) examine the association between unmet health care needs and satisfaction with health care services among neurological patients in Canada, and (3) contrast patient satisfaction between physician care and hospital care among Canadians with NCs.

## Methods

### Study participants and data sources

Data were extracted from the 2010 Canadian Community Health Survey - Annual Component (CCHS − 2010). This cross-sectional survey collected population-wide information on health status, health care utilization, and health determinants of Canadians aged 12 + living in private households in all provinces and territories [26]. Persons living on Crown lands or Indian Reserves, those dwelling in institutions, or certain remote regions, as well as full-time members of the Canadian Forces, are excluded from this survey [26]. Approximately half the interviews were conducted in person using computer-assisted personal interviewing (CAPI) and the other half were conducted over the phone using computer-assisted telephone interviewing (CATI) [26]. The overall person-level survey response rate was 88.6% and the combined response rate was 71.5% at the national level. Statistics Canada’s research ethics board approved the original survey [26].

**Fig. 1:**
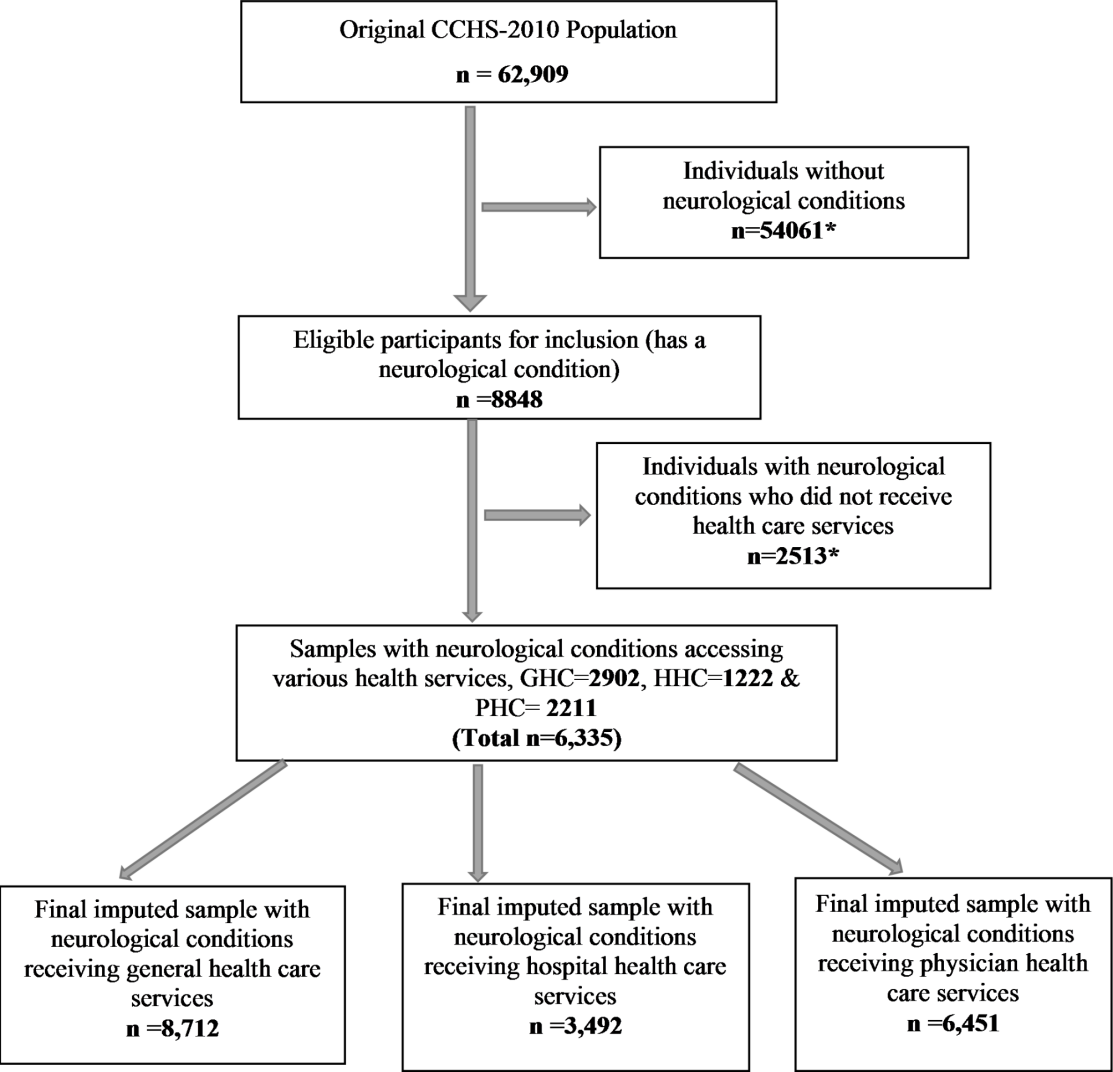
Restriction criteria employed to obtain the sub-sample in this study. * Excluded from the analysis

The CCHS-2010 was used due to its one-year unique common content on health care utilization: unmet health care needs (UCN) and neurological conditions and the optional content on patient satisfaction [26]. Residents of Ontario with NCs who received health care services completed the module on patient satisfaction and provided content on unmet health care needs were assessed. The population of 10,819,146 in Ontario in 2010 represented a little over one-third of the Canadian population in that year. The views of those respondents should provide good insight into the concerns of Canadians with NCs. Therefore, an imputed subsample of 6335 respondents with NCs was used for this study. From that number, 2902 who received general health care services, 1222 who received hospital services, and 2211 who received physician services within twelve months leading up to data collection were selected. Age categories 12–44 years were grouped to protect anonymity, due to the small sample size of the study population, and very few people in the youngest age categories reported NCs and unmet health care needs. This study was carried out in accordance with the relevant national/institutional guidelines and regulations. Figure 1 below demonstrates the restriction criteria used to obtain the subsample from the original sample.

### Derivation of neurological conditions variable

Neurological conditions in the CCHS-2010 sample were derived from responding “yes” to having a neurological condition: Alzheimer’s disease or dementia, Parkinson’s disease, multiple sclerosis, epilepsy, cerebral palsy, amyotrophic lateral sclerosis, Huntington’s disease, stroke effects, Tourette’s syndrome, dystonia, muscular dystrophy, spina bifida, brain injuries, spinal cord injury, brain and spinal cord tumors, hydrocephalus, and migraine headaches.

### Assessment of patient satisfaction (outcome)

Patient satisfaction as our outcome of interest was defined according to satisfaction with health care in general (health care services from any health care provider including ophthalmologists, dentists, and other allied health professionals and home care); hospital (health care services at a hospital, for any diagnostic or day surgery service, overnight stay, or as an emergency room patient); and physician services (health care services from a family doctor (general practitioner), and other physicians (medical specialist). Respondents answered the following questions: “Overall, how satisfied were you with the way health care services were provided?” “How satisfied were you with the way hospital services were provided?” “How satisfied were you with the way physician care was provided?” Responses for the levels of satisfaction with the various types of health care services were ordinal and coded by categories: 1 = very satisfied, 2 = somewhat satisfied, 3 = neither satisfied nor dissatisfied, 4 = somewhat dissatisfied, and 5 = very dissatisfied. For each patient satisfaction variable (general health care, hospital, and physician), categories 1 and 2 were collapsed and recoded as “satisfied” = 1, while categories 3–5 were collapsed and recoded as “dissatisfied” = 0.

### Primary predictor (self-perceived unmet health care needs)

We examine the relationship between self-perceived unmet health care needs and patient satisfaction. Self-perceived unmet care need was identified in the CCHS-2010 by the question, “During the past 12 months, was there ever a time when you felt that you needed health care but you didn’t receive it?” Responses were coded, “yes” = 1 and “no” = 0. For this variable, reasons for indicating unmet care needs include (1) unavailability of care – waiting time too long, care not available when requested, care not available in the area, the doctor didn’t think the care was necessary (2) unacceptability of care – dislike doctor/afraid, language problems, didn’t know where to go (3) inaccessibility –cost (4) personal choice – too busy, didn’t get around to it/didn’t bother, felt it would be inadequate, decided not to seek care, and personal/family responsibilities.

### Covariates

**Fig. 2:**
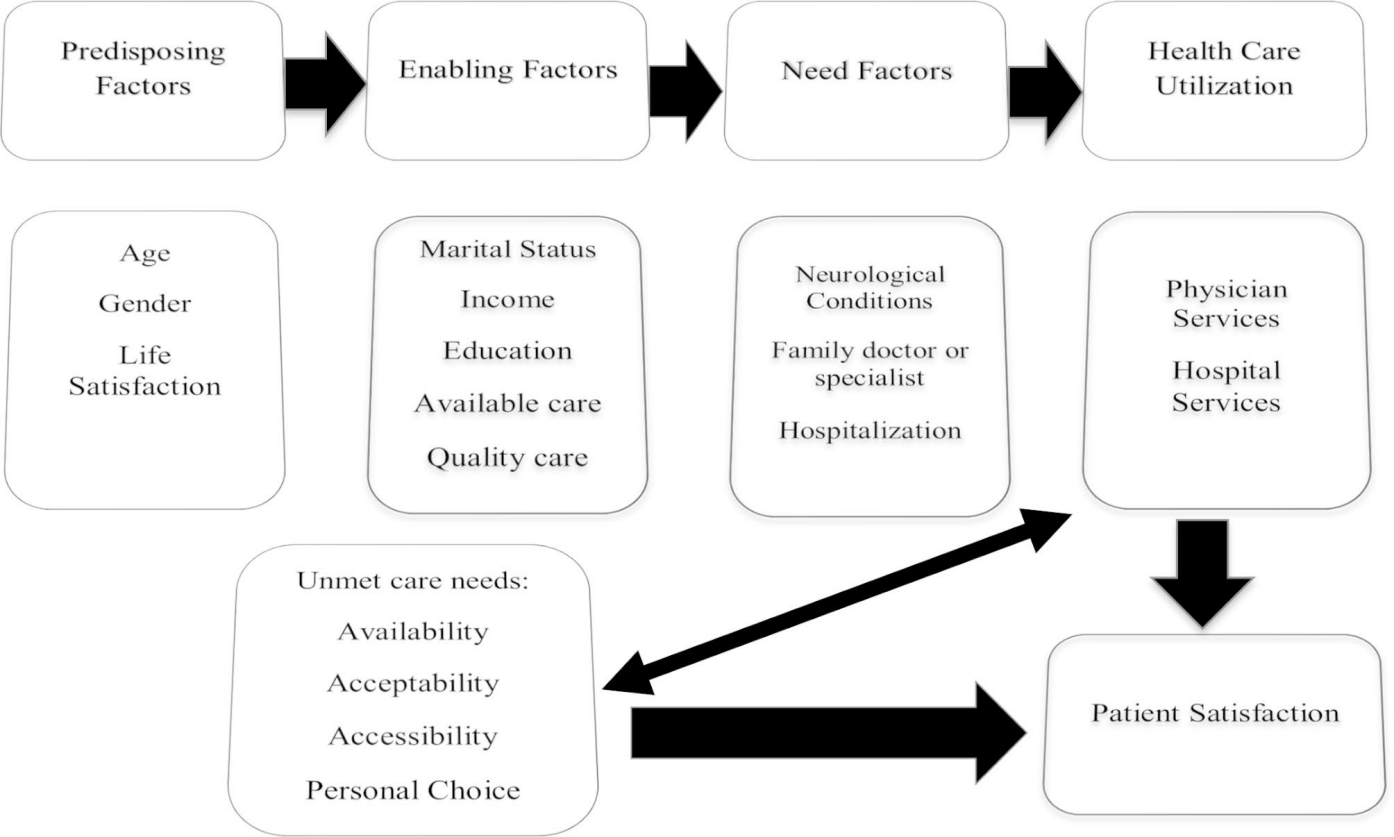
Research model of health care utilization in current study based on Andersen’s health behavior model

Other sociodemographic covariates assessed were: *age* (< 45, 45–64, 65–79, 80 + years), *sex* (“male” vs “female”), *marital status* (“married”, “common-law”, “widowed/divorced/separated”, “single/never married”), *level of education* (“less than secondary”, secondary graduation”, “some post-secondary education”, “post-secondary graduation”), *total personal income from all sources* (≤ 19,999, 20,000–39,999, 40,000–69,999, 70,000 or more), *satisfaction with life in general* (“dissatisfied”, “very satisfied”, “satisfied”, “neither satisfied nor dissatisfied”). Ratings of availability of provincial health care were assessed as: *general health care* (“poor”, “fair”, “good”, “excellent”); *hospital services* (“poor”, “fair”, “good”, “excellent”); and *physician services* (“poor”, “fair”, “very good”). Rating of quality of care received: *general health care* (“poor”, “fair”, “good”, “excellent”); *hospital services* (“poor”, “good”, “excellent”); and *physician services* (“poor”, “good”, “excellent”). Type of patient at most recent visit (“admitted overnight”, “outpatient”, “ER patient”). Type of physician seen at most recent visit (“family doctor” vs “specialist”). Categories of “do not know”, “refusal” and “not stated” were treated as missing values. Our study is grounded on Andersen’s health behavior model as shown in (Fig. 2) below.

### Statistical analysis

Statistical analysis was completed using STATA version 14. Sampling weights were applied to account for the survey design. Descriptive statistics were tabulated for the main exposure variable, outcome variable, and covariates as well as socio-demographic factors (age, gender, marital status, education, and personal income) among those with NCs. To account for missing data, and prevent loss of information and selection bias, chained iterations of multiple imputations were conducted [27]. All missing values were retrieved and included in the final model-building process.

Logistic regression was used to estimate the association between predictor variables and general life satisfaction due to the small sample size and because the assumptions for ordered logistic regression were violated. The outcome variable categories were collapsed and logistic regression was conducted because generalized ordered logistic regression models did not converge in the model-building process. Univariate logistic regression models were utilized to examine the association between self-perceived unmet care needs, other predictors/covariates, and satisfaction with health care services. Unadjusted odds ratios and 95% confidence intervals (CI) and p-values were calculated. Predictors/covariates with unconditional p-values ≤ 0.20 were retained for use in the multivariate model-building phase of analysis [4]. In the multivariate model building process, variables with p-values > 0.05 were individually eliminated in a sequence of descending p-values, using a manual backward elimination strategy. Variables with significant p-values ≤ 0.05 were retained in the final model. All variables of interest which were manually eliminated due to insignificant p-values were checked for confounding and retained when they altered the coefficients for the exposure of interest by > 20%. Any variable with an initial insignificant p-value that was eliminated at the univariate analysis stage was assessed for interaction. A likelihood ratio test assessed the overall significance of our logistic regression model.

## Results

### Characteristics of the study population – individuals with neurological conditions

Analysis for this study was limited to the imputed data of the original subsamples of 2902, 1222, and 2211 individuals with NCs who received general health care services, hospital services, and physician services respectively. Table 1 below demonstrates the demographic characteristics of the study population for all three study samples. The total number of cases varies due to missing values.

There is little variation in socio-demographic characteristics across subsamples. Over two-thirds of the subsamples were females (67.8, 68.8, and 70.2% respectively) and under 65 years of age (71.5, 67.2, 71.9% respectively). A little under half of the respondents reported postsecondary graduation (45.2, 45.3, and 47.4% respectively). Less than half of the respondents in all samples were married (40.4, 38.7, and 40.1% respectively), while just under half earned ≤$19,999 annually (43.1, 44.6, and 43.3%) and under 20% in each sample reported unmet health care needs.

**Table 1:** Sociodemographic characteristics of study samples by health care services use: general (8,712), hospital (3,492) and physician (6,451) services

Characteristics	General Health Care Services	Hospital Services	Physician Services
	n(%)*	n(%)*	n(%)*
**Age categories, years**			
≤44 years	3,507 (40.2)	1,242 (35.6)	2,553 (39.6)
45 to 64	2,725 (31.3)	1,103 (31.6)	2,086 (32.3)
65 to 79	1,636 (18.8)	758 (21.7)	1,125 (17.4)
80 and above	844 (9.7)	389 (11.1)	687 (10.7)
**Sex**			
Male	2,804 (32.2)	1,091 (31.2)	1,925 (29.8)
Female	5,908 (67.8)	2,401 (68.8)	4,526 (70.2)
**Marital status**			
Single	2,583 (29.7)	954 (27.4)	1,843 (28.6)
Married	3,515 (40.4)	1,349 (38.7)	2,583 (40.1)
Common-law	413 (4.7)	193 (5.5)	301 (4.7)
Widowed/separated/divorced	2,193 (25.2)	992 (28.4)	1,717 (26.6)
**Educational level**			
Less than secondary	2,517 (29.0)	1,033 (29.7)	1,774 (27.6)
Secondary grad	1,569 (18.1)	543 (15.6)	1,116 (17.4)
Other post-secondary	666 (7.7)	326 (9.4)	485 (7.6)
Post-secondary graduation	3,916 (45.2)	1,577 (45.3)	3,046 (47.4)
**Income status**			
<=19,999	3,541 (43.1)	1,468 (44.6)	2,634 (43.3)
20,000–39,999	2,376 (29.0)	1,037 (31.5)	1,828 (30.1)
40,000–69,999	1,548 (18.9)	536 (16.3)	1,074 (17.7)
≥ 70,000	735 (9.0)	252 (7.6)	544 (8.9)

*Values and percentages included imputed data

The results in Table 2 below describe the variables associated with health care services received by the respondents. Over two-thirds of the respondents were satisfied with general, hospital and physician services (83.9, 81.1, and 91.2% respectively). Less than half of the respondents felt they received excellent general, hospital, and physician health care (38.3, 45.9, and 54.6% respectively). Less than half of the respondents who received hospital services were outpatients (39%) while the majority received physician services from a family doctor (82.3%) (Table 2).

**Table 2:** Description of variables associated with utilization of health care services: general (8,712), hospital (3,492) and physician (6,451) services

Variables	General Health Care Services N( %)*	Hospital Services N(%)*	Physician Services N(%)*
**Unmet health care needs**			
No	7,329 (84.2)	2,797 (80.2)	5,249 (81.4)
Yes	1,375 (15.8)	691 (19.8)	1,197 (18.6)
**General life satisfaction**			
Dissatisfied	590 (6.8)	279 (8.0)	434 (6.7)
Very satisfied	2,787 (32.2)	1,012 (29.2)	2,007 (31.3)
Satisfied	4,396 (50.7)	1,822 (52.5)	3,342 (52.1)
Neither satisfied/dissatisfied	895 (10.3)	359 (10.3)	633 (9.9)
**Rating of availability of Provincial care**			
Poor	1,218 (14.0)	537 (15.5)	845 (13.2)
Fair	2,182 (25.2)	923 (26.6)	1,647 (25.6)
Good	3,884 (44.8)	1,381 (39.7)	3,928 (61.2)^1^
Excellent	1,392 (16.0)	633 (18.2)	
**Quality of Care Received**			
Poor	299 (3.4)	595 (17.0)	694 (10.8)
Fair	1,072 (12.3)		
Good	3,993 (45.9)	1,293 (37.1)^2^	2,233 (34.6)^2^
Excellent	3,336 (38.3)	1,599 (45.9)	3,517 (54.6)
**Patient Satisfaction**			
Dissatisfied	1,396 (16.1)	660 (18.9)	568 (8.8)
Satisfied	7,299 (83.9)	2,827 (81.1)	5,875 (91.2)
**Most recent patient**			
Outpatient	-	1,363 (39.0)	-
Admitted Overnight	-	817 (23.40)	-
ER Patient	-	1,312 (37.6)	-
**Physician Type**			
Family Doctor	-	-	5,303 (82.3)
Specialist	-	-	1,144 (17.7)

^1^ Good and excellent categories collapsed to very good

^2^ Fair and good categories collapsed into good

* Results included inputted values

### Characteristics associated with patient satisfaction with general health care, hospital, and physician services (multivariate analysis)

Table 3 demonstrates the results of the final multivariate logistic regression models for patient satisfaction with adjusted predictor and/or covariate variables. We found self-perceived unmet health care needs to be a strong negative predictor for patient satisfaction across all health care services. For those with self-perceived unmet needs, the greatest dissatisfaction was associated with physician services (OR = 0.29, p = 0.005), followed by hospital services (OR = 0.41, p = 0.006) and general health care services (OR = 0.59, p = 0.024), when compared to those without unmet health care needs. Conversely, quality and availability of care were significant protective predictors of patient satisfaction across all health care services. When compared to those who received poor quality care, the odds of patient satisfaction (general health care services, 237.60, p < 0.001; hospital services,166.99, p < 0.001; and physician services, 176.4, p < 0.001) were highest across all services among those who received excellent quality care; with those receiving general health services most likely to be satisfied with quality care: fair (OR = 6.15, p = 0.002), good (OR = 36.37, p < 0.001) and excellent (OR = 237.60, p < 0.001) (Table 3). The odds of patient satisfaction across all health services were higher with the increasing availability of care. When compared to poor availability of care, the odds of patient satisfaction were highest among those who reported excellent care availability across health care services in general (OR = 4.45, p < 0.001) and hospital services (6.30, p < 0.001), with those receiving hospital services increasingly satisfied with levels of care availability: fair (OR = 2.77, p = 0.011), good (OR = 3.90, p < 0.001) and excellent (OR = 6.30, p < 0.001).

Education was a negative predictor of patient satisfaction among those who received general health services with higher levels of education being more dissatisfied with care, [(secondary graduate, OR = 0.62, p = 0.126); (other post-secondary, OR = 0.36, p = 0.004); and post-secondary graduate, OR = 0.54, p = 0.050)] and those who received physician services [(secondary graduate, OR = 0.32, p = 0.010); (other post-secondary, OR = 0.26, p = 0.019); and post-secondary graduate, OR = 0.28, p = 0.005)]. Post-secondary graduation provided reduced odds of being satisfied with hospital services compared to those with the lowest levels of education (Table 3).

Physician type seen and most recent type of patient during last health care services were also predictors of patient satisfaction with physician and hospital services respectively. Respondents who received specialist care were 47% less likely (OR = 0.47, p = 0.106) to be satisfied with physician services than those who received care from a family doctor. Patients who were admitted overnight were more likely (OR = 1.20, p = 0.660) to be satisfied with hospital services than outpatients, while ER patients were significantly less likely (OR = 0.39, p = 0.007) to be satisfied with hospital services than both outpatients and overnight patients.

**Table 3:** Multivariate analysis of predictors of patient satisfaction with general (8,712), hospital (3,492), and physician (6,451) services

Variables	General Health Care Services*		Hospital Services*		Physician Services*	
	OR, 95% CI	p-Value	OR, 95% CI	p-Value	OR, 95% CI	p-Value
**Age categories, years**						
≤44 years	Reference		Reference		Reference	
45 to 64	1.80 (0.98–3.32)	0.059	2.02 (0.83–4.91)	0.120	1.14 (0.51–2.57)	0.747
65 to 79	1.24 (0.59–2.59	0.576	0.39 (0.13–1.17)	0.092	0.75 (0.27–2.08)	0.585
80 and above	2.57 (0.97–6.86)	0.059	0.48 (0.11–2.13)	0.334	0.73 (0.23–2.29)	0.593
**Sex**						
Male	Reference		Reference		Reference	
Female	1.32 (0.80–2.17)	0.276	1.12 (0.53–2.37)	0.764	0.62 (0.32–1.19)	0.152
**Marital status**						
Single	Reference		Reference		Reference	
Married	1.39 (0.78–2.50)	0.264	0.55 (0.19–1.61)	0.278	0.62 (0.31–1.21)	0.160
Common-law	1.31 (0.67–2.58)	0.427	0.69 (0.23–2.10)	0.514	1.21 (0.42–3.53)	0.727
Widowed/separated/divorced	1.48 (0.76–2.89)	0.254	0.68 (2.01–2.27)	0.524	1.06 (0.41–2.71)	0.603
**Educational level**						
Less than secondary	Reference		Reference		Reference	
Secondary graduate	0.62 (0.38–1.14)	0.126	2.00 (0.73–5.47)	0.177	**0.32** (0.13–0.76)	0.010
Other post-secondary	**0.36** (0.18–0.72)	0.004	2.81 (0.94–8.40)	0.065	**0.26** (0.09–0.80)	0.019
Post-secondary graduate	0.54 (0.29–1.00)	0.050	0.98 (0.42–2.28)	0.967	**0.28** (0.11–0.67)	0.005
**Income status**						
≤ 19,999	Reference		Reference		Reference	
20,000–39,999	0.73 (0.41–1.30)	0.281	1.49 (0.58–3.80)	0.404	1.57 (0.69–3.54)	0.278
40,000–69,999	1.54 (0.75–3.17)	0.242	1.04 (0.36–3.00)	0.939	1.06 (0.33–3.46)	0.917
≥ 70,000	0.90 (0.39–2.12)	0.817	0.33 (0.11–0.97)	0.045	1.17 (0.38–3.63)	0.783
**Unmet health care needs**						
No	Reference		Reference		Reference	
Yes	**0.59** (0.37–0.93)	0.024	**0.41** (0.21–0.77)	0.006	**0.29** (0.13–0.69)	0.005
**General life satisfaction**						
Dissatisfied	Reference		Reference		Reference	
Very satisfied	**2.15** (1.03–4.49)	0.041	1.56 (0.45–5.41)	0.481	2.53 (0.88–7.26)	0.084
Satisfied	1.80 (0.92–3.53)	0.085	0.77 (0.25–2.33)	0.642	1.24 (0.46–3.37)	0.668
Neither satisfied nor dissatisfied	1.29 (0.60–2.76)	0.510	0.46 (0.13–1.69)	0.244	1.60 (0.57–4.52)	0.372
**Availability of provincial care**						
Poor	Reference		Reference		Reference	
Fair	**1.72** (1.03–2.87)	0.039	**2.77** (1.27–6.05)	0.011	1.25 (0.54–2.93)	0.592
Good	**3.18** (1.78–5.68)	< 0.001	**3.90** (1.92–7.92)	< 0.001	1.10 (0.44–2.75)	0.833
Excellent	**4.45** (1.76–11.25)	< 0.001	**6.30** (2.35–16.86)	< 0.001		
**Quality of care received**						
Poor	Reference		Reference		Reference	
Fair	**6.15** (2.00–18.94)	0.002				
Good	**36.37** (12.09–109.44)	< 0.001	**35.61** (18.71–67.78)	< 0.001	**26.78** (13.36–53.69)	< 0.001
Excellent	**237.60** (70.43–801.52)	< 0.001	**166.99** (67.91–410.64)	< 0.001	**176.45** (63.89–487.30)	< 0.001
**Most recent patient**						
Outpatient			Reference			
Admitted Overnight			1.20 (0.53–2.72)	0.660		
ER Patient			**0.39** (0.20–0.77)	0.007		
**Physician type**						
Family Doctor					Reference	
Specialist					0.47 (0.18–1.18)	0.106

*Results included imputed values

Significant values are marked in **bold print**

Hosmer-Lemeshow (χ^2^) and p-values for General health care services (H-L: 13.74; p-value = 0.0888); Hospital services (H-L: 29.80; p-value = 0.0002); Physician services (H-L: 19.15; p-value = 0.0141)

**Fig. 3:**
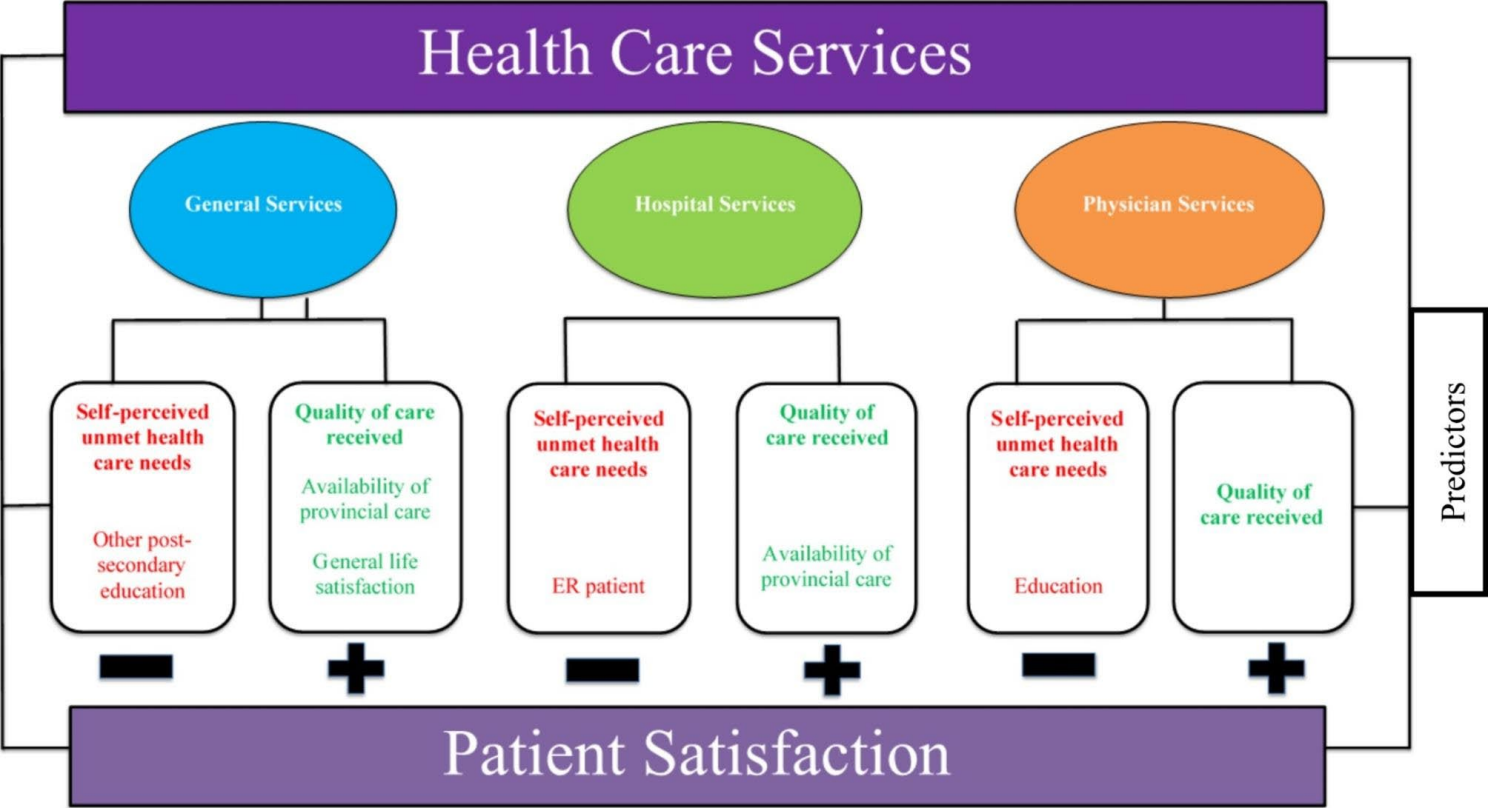
Summary of significant associations between health care services and patient satisfaction among individuals with neurological conditions

In summary, quality of care is strongly and positively associated with patient satisfaction across all health services. Other significant positive predictors of patient satisfaction are the availability of provincial care, quality of care received, and being very satisfied with life in general. The common significant negative predictor of patient satisfaction across all healthcare services is self-perceived unmet health care needs. Post-secondary education (general health services and physician services), and being an ER patient most recently (hospital services) also demonstrated significant negative associations with patient satisfaction. (Fig. 3).

## Discussion

The major findings of our study can be summarized by Anderson’s health behavior model predicting health care utilization factors in our model: predisposing (age, gender, and general life satisfaction), enabling (marital status, income, education, availability of health care, and quality of care), and need factors (neurological patients’ use of general health care services, hospital and physician services) and patient satisfaction. One enabling factor, quality of received care, demonstrated a strong positive association with patient satisfaction with all health care services received in this study, while another, availability of provincial care, was positively associated with patient satisfaction with general health care and hospital services. One predisposing factor, general life satisfaction, was positively associated with patient satisfaction with general health care services. On the other hand, identified as a disabling factor, self-perceived unmet health care needs commonly reduced the odds of patient satisfaction with the need factors, health care services in general, physician and hospital services. Education was also deemed a disabling factor, with all levels negatively associated with patient satisfaction with physician care. The need factor, ER services, was negatively associated with patient satisfaction with hospital services.

Of particular interest is the relationship between patient satisfaction and the predisposing factors of health care utilization, General Life Satisfaction (GLS) represents the quality of life in several studies [28–30]. Our finding that GLS was positively associated with patient satisfaction with general health care services is consistent with that of other studies that reported satisfaction in life domains as positively associated with patient satisfaction [31]. While GLS influencing health-related quality of life may be positively associated with patient satisfaction with general health care services, significant decreases in health-related quality of life among people living with long-term neurological conditions have been reported in other studies [32–36]. This positive association between increased levels of GLS and greater odds of patient satisfaction among neurological patients may result in increased health-related quality of life due to the enabling factors, availability, and quality of care.

Our study found that while availability and quality of care were positive predictors of patient satisfaction across all health services, it was not significantly associated with patient satisfaction with physician services. Availability and quality of care are important predictors of health-related quality of life as satisfied patients are more likely to comply with treatment, demonstrate positive health behaviors, and register improved health outcomes [37, 38]. Consistent with our study, one other study found that quality of care was associated with high levels of patient satisfaction among neurological patients [39]. The quality of care in that study referred to the early connection between patients and neurologists and education and advice on living with neurological conditions [39]. A similar study of neurological patients, found high patient satisfaction with coordination of safe, compassionate, and multiple health care services for those with mobility challenges [40], supporting our finding that when health care services are available, the odds of neurological patient satisfaction are increased.

The association of unmet health care needs, patient satisfaction with health care services, and health-related quality of life have been reported in earlier studies [18, 41]. Patient satisfaction is positively associated with health-related quality of life [42–44]. One particular study that examined the relationship between unmet health care needs and health-related quality of life among patients with multimorbidity [45], found that the presence of unmet health care needs was associated with lowered health-related quality of life. It may be deduced from this study that self-perceived unmet health care needs are associated with health-related quality of life among neurological patients, though we did not predict the direction of that association.

Higher education levels and hospital admission through the emergency room (ER) were associated with decreased patient satisfaction in our study. This is consistent with findings of other studies [46, 47], one of which suggests that health care providers may create a better patient experience through increased communication or more active referral of ER patients to patient representatives [46]. One other study found that the highest level of education strongly predicted favorable satisfaction with communication with doctors [48]. This suggests that the negative association between the highest levels of education and patient satisfaction among individuals with NCs in our study may be due to communication needs not being met.

The association between ER care in hospitals and lower patient satisfaction in our study may be explained by a reduction in one or more of the components of patient satisfaction proposed by Mollaoğlu and Çelik [49]: guidance, debriefing, paying attention and being kind, having empathy, providing psychosocial support, speed of service, timing, proficiency, and overall quality. In addition, the severity of a patient’s condition [50] and the stress of a neurological patient being in the ER [49] may negatively influence patients’ level of satisfaction with emergency services. Finally, our study demonstrated an association between availability of care and lower odds of patient satisfaction among ER neurological patients who received hospital services. This may be indicative of decreased availability of care– waiting time too long, healthcare not available when requested, and healthcare not available in the area (elements of unmet health care needs reported in the CCHS-2010) [26].

### Strengths and limitations of the study

One strength of this study is that it supports the finding that unmet health care needs are a risk factor for decreased patient satisfaction among neurological patients and that available and quality care are positive predictors of patient satisfaction across health services. Other strengths include the use of a nationally representative survey of the Canadian population with relatively high participation rates allowing for generalization of study findings; and the provision of information on specific health care services, i.e., general health care services, hospital and physician services that may vary in their impact on neurological patients.

Limitations are noted. Persons living on lands designated as Indian Reserves or by the Crown, those dwelling in institutions, or certain remote regions as well as full-time members of the Canadian Forces are excluded from this survey. The representation of those residing in institutions would have been valuable to this study. This exclusion and the possible selection bias of individuals who were functionally capable of responding to the questionnaires are limitations that may impact the generalizability of the study. The use of data from optional modules causes a reduction in the sample size, decreasing the generalizability of the findings to the entire population. The relatively small sample size did not facilitate subgroup analysis by types of neurological conditions. Types of unmet health care needs and neurological conditions were not specified. The severity of disease conditions was not measured, making it difficult to address patient satisfaction or targeted interventions within groups of neurological conditions with specific unmet health care needs. Finally, the study could not perform a stratified analysis by income differences (< $20,000 annual income versus $40,000 + annual income) due to the small sample size of the study population in some categories and the need to meet anonymity, confidentiality, and data release rules of the research data centre. This is important in determining the potential influence of income on life satisfaction among neurological patients.

## Conclusion

Self-perceived unmet health care needs are a common significant negative predictor of neurological patients’ satisfaction across health care services. Future studies on predictors of neurological patients’ satisfaction with health care services should focus on specific unmet health care needs and different neurological conditions. Neurological patients are known to report unmet health care needs and experience barriers to care, limiting their quality of life. Our study emphasizes that the availability and accessibility of care for neurological patients increased the satisfaction with health care services in general as well as physician and hospital services.

## Data Availability

The data that support the findings of this study are from the CCHS-2010 anonymized Master Files and are not publicly available. Data can be accessed using Statistics Canada confidential microdata files (Master data files) through a Research Data Centre only (https://crdcn.org). Access can be arranged directly through DLI enquiries: statcan.maddli-damidd.statcan@canada.ca.

## Abbreviations

AD: Alzheimer’s disease.
CCHS: Canadian Community Health Survey.
CRDCN: Canadian Research Data Centre Network.
ER: Emergency Room.
GLS: General Life Satisfaction.
NCs: Neurological conditions.
